# Agreement between intraocular pressure measurement using Goldmann applanation tonometry with and without fluorescein in consecutive Ghanaian patients

**DOI:** 10.4314/gmj.v59i3.4

**Published:** 2025-09

**Authors:** Charles Darko-Takyi, Youfegan B Mathurin, Emmanuel Essien, Sandra Owusu, Osei A Yaw, Kwame O Osei, Selina Holdbrook, Andrew Owusu-Ansah

**Affiliations:** 1 School of Optometry and Vision Science, College of Health and Allied Sciences, University of Cape Coast, Ghana; 2 University of Cape Coast Eye Clinic, Cape Coast, Ghana; 3 Our Lady of Grace Catholic Mission Hospital, Bremen Esikuma, Ghana; 4 Korle-Bu Teaching Hospital Eye Centre, Accra, Ghana

**Keywords:** Intraocular Pressure, Goldmann Applanation Tonometry, With fluorescein, Without Fluorescein, Ghanaian patients

## Abstract

**Objective:**

The interchangeability of Goldmann Applanation Tonometry (GAT) with or without fluorescein requires clarification among African populations. The present study aimed to determine the agreement between IOP measurement using GAT with and without fluorescein in consecutive patients in Ghana.

**Design:**

A prospective cross-sectional clinic-based study design.

**Setting:**

The study was conducted at the Ophthalmology and Optometry units of the UCC Eye Clinic, Cape Coast, Ghana.

**Participants:**

A convenient sample of 104 consecutive patients who visited the clinic. The participants were randomised into two groups, where those in the first had their IOPs first measured without fluorescein and later with fluorescein on GAT; participants in the second group had their IOPs first measured with fluorescein and later without fluorescein after a 10-minute washout period. The central corneal thickness was measured using the Anterior Segment model of Zeiss Cirrus HD-OCT.

**Results:**

There was no agreement between the unadjusted IOP measures with and without fluorescein with GAT using the Bland Altman analysis (p < 0.0001, Lower limit: -0.9433, Upper limit: 6.8914; 95% CI = -1.6097 to -.02769). There were no statistically significant differences in gender between unadjusted IOP measurements with (Mann Whitney U test, p = 0.110) and without (Mann-Whitney U test, p = 0.083) fluorescein.

**Conclusion:**

Unadjusted IOP measurement without fluorescein was approximately 3 mmHg significantly lower than with fluorescein. We recommend practitioners maintain the gold standard GAT with fluorescein to guide accurate glaucoma diagnosis and management decision-making.

**Funding:**

Self-funded

## Introduction

Glaucoma is a bracket of progressive optic neuropathies identifiable by a degeneration of retinal ganglion cells and retinal nerve layers that contributes to the alteration of the optic nerve head^1^,^2^ and where intraocular pressure (IOP) is a major essential modifiable risk factor^3^,^4^. Glaucoma is reported as the second principal cause of blindness globally.^5^ Some over 80 million people globally were estimated to have glaucoma by the year 2020 6 and this number is projected to increase to 111.8 million by 2040.^7^ Apart from increased IOP, other leading risk factors include age, gender, refractive status, family history, use of steroids, systemic hypotension, and hypertension.^8^ Management and control of IOP are the most effective interventions covering the various classes of glaucoma. IOP elevation is highly associated with glaucoma onset and development to the detriment of the nerve fibre layer and consequent visual field loss.^9^ Therefore, accurate and precise measurement of IOP coupled with other diagnostic tools such as Optical coherence tomography and perimetry will culminate in proper diagnosis and management of glaucoma salvaging its repercussions.^10^,^11^

Goldmann applanation tonometry (GAT) with fluorescein is the gold standard in measuring IOP 12,13,14; however, some authors^12^,^13^,^15^,^16^,^17^,^18^,^19^ have experimented with the use of GAT without the use of fluorescein to determine if it gives comparable results to the gold standard. Many practitioners establish the use of fluorescein to improve visualisation of mires when measuring the IOP level 15,20; other practitioners also emphasise the use of fluorescein in GAT as an opportunistic tool to assess the integrity of the cornea before checking the IOP.^16^ That notwithstanding, many other practitioners check IOP without the use of fluorescein, bringing into perspective its clinical drawbacks, which include no clear-cut clinical standardisation of fluorescein, which may erroneously influence IOP measurements in addition to a burning sensation, allergic reactions, pruritus, susceptibility to infection, high cost, and less availability of fluorescein dye.^12^,^15^,^16^ This has led to a well-established debate on the outcome of results revealed in this area of study, as the literature gives conflicting ideas among different populations. According to studies,^13^,^15^,^17^,^18^,^19^, some stipulations imply significantly lower results of IOP measurement using the GAT technique without fluorescein than with fluorescein. That notwithstanding, other studies^12^,^16^ also reported an antagonistic view, which posits that there is no clinically significant difference in the measurement of IOP with GAT with and without the use of fluorescein. No study, however, has investigated the relationship between GAT measurement of IOP with and without fluorescein in the West African population.

Therefore, this study sought to investigate the agreement between measuring IOP with and without fluorescein with GAT in a Ghanaian population, thereby guiding practitioners in the proper diagnosis and management of glaucoma. It also aimed to compare the adjusted (correction factor) and unadjusted IOPs with and without fluorescein using GAT in a Ghanaian population.

## Methods

### Ethical consideration

The study was approved by the Institutional Review Board (IRB) of the University of Cape Coast, Ghana (Reference: UCCIRB/CHAS/2023/35) and followed the tenets of the Declaration of Helsinki on the use of human subjects for research. Written informed consent was obtained from participants before the commencement of the study.

### Study Design, population, and sampling

A clinic-based cross-sectional study design utilising a convenience sampling technique was used. All clients aged 18 years and above who consecutively visited the University of Cape Coast Eye Clinic, Ghana, within the study period (October 2023 to December 2023), who met the study's requirements and gave consent, were enrolled as participants.

### Data collection procedure

#### Preliminary examination

Participants underwent distance visual acuity examination using the Snellen chart at 6m. A slit lamp assessment of the anterior segment was then performed. Dry eyes assessment was performed using the tear break-up time technique.

#### Exclusion criteria

Participants with corneal abnormalities, acute eye inflammation or infections, bilateral blindness, eye surgery, ocular trauma, and dry eye were excluded from the study.

#### Measuring central corneal thickness

The central corneal thickness (CCT) of each participant who satisfied the inclusion criteria was taken using the Anterior Segment module of the Optical Coherence Tomography (Zeiss Cirrus 500 HD-OCT Model, Carl Zeiss Meditec, Dublin, CA, USA).

#### IOP measurement with and without fluorescein

Participants who satisfied the inclusion criteria were randomised into groups A and B; the randomisation was done such that participants were made to pick folded papers with inscriptions A or B indicated in them from an opaque container. Participants in group A had their IOP first measured with GAT without fluorescein and subsequently with fluorescein after a rest phase of about 10 minutes. Participants in group B had their intraocular pressure measured first with fluorescein and, following a washout phase of 10 minutes, without fluorescein. The IOPs were each measured after a topical anaesthetic (proparacaine hydrochloride 0.5%) had been instilled. Participants' IOP using GAT with fluorescein was measured using 1 drop of fluorescein sodium dye 2% v/w on the eye and observing for mires under the cobalt blue filter of the slit lamp biomicroscope. Participants' IOP using GAT without fluorescein was measured using the white light illumination of the slit lamp. The IOPs with and without fluorescein were measured in one eye only for each participant. The selection of right and left eyes for IOP measurement was randomised; half of the participants who picked folded papers labelled right eye and left eye had their IOPs measured with and without fluorescein for their right and left eyes, respectively. To prevent inter-examiner variability in the results, IOP measurements were taken by one examiner only. Unadjusted IOP measures with and without fluorescein for each participant were noted and adjusted for CCT using the Ehlers correction factor [Ehlers Adjusted IOP = Measured IOP + [0.071 × (545 − CCT] nomogram^21^ and the Linear model's formula^22^ [Adjusted IOP = Measured IOP − (CCT-545) / 50 × 2.5mmHg]. CCT of 545 µm was used in the Linear model's formula for easy comparison, as it is the standard used in the Ehlers correction factor; 545 µm is also within the range (of CCT) determined for the study population (Table 1).

**Table 1:** Descriptive statistics of Unadjusted and Adjusted (Ehlers and Linear Model) with and without fluorescein on GAT

	IOP measurements with fluorescein			IOP measurements without fluorescein			CCT

	Unadjusted	Adjusted	Adjusted	Unadjusted	Adjusted	Adjusted	Um
	(f GAT)	(Ehlers)	(Linear)	(nf GAT)	(Ehlers)	(Linear)	
**Mean**	14.34	15.29	15.10	11.36	12.34	12.12	529.80
**+/- SD**	3.33	3.03	3.01	3.17	3.05	2.97	31.07
**Maximum**	32.60	33.60	33.65	25.00	26.00	26.05	601.00
**Minimum**	7.60	9.60	10.10	6.00	4.30	4.80	471.00
**Range**	25.00	24.00	23.55	19.00	21.70	21.25	130.00
**5%**	10.00	10.78	10.86	7.08	7.40	7.54	478.50
**25%**	12.00	13.63	13.21	8.70	10.70	10.41	508.25
**50%**	13.85	15.00	14.75	11.30	12.30	12.10	524.50
**IQR**	4.60	3.38	3.65	4.30	3.30	3.47	42.00
**75%**	16.65	17.00	16.86	13.00	14.00	13.89	549.75
**95%**	19.00	19.00	18.89	16.93	16.60	16.13	590.25
**Shapiro Wilk**	0.0001	0.0001	0.0001	0.0001	0.0001	0.0001	0.185
**p-values**							

f GAT: Unadjusted IOP measurement with fluorescein.

nf GAT: Unadjusted IOP measurement without fluorescein.

GAT – Goldmann applanation tonometry

### Data analysis

Data was analysed using the IBM SPSS version 22.0. The Wilcoxon signed-rank test was used to determine the difference between the two techniques. Spearman correlation was used to determine the correlation between the two techniques and other continuous variables. The agreement between measuring intraocular pressure with and without fluorescein with GAT was investigated using Bland-Altman analysis. Statistical significance was set at a p-value of ≤ 0.05.

The total number of participants enrolled in the study was 153 of which 49 were excluded with corneal abnormalities (8), acute eye inflammation or infections (13), eye surgery (2), ocular trauma (1), and dry eye (23). Two (2) participants were uncooperative with the GAT assessment and 104 completed all assessments. Of the 104 participants (104 eyes), fifty-two, 52 (50%) were males and fifty-two, 52 (50%) were females. Their age ranged from 19 to 29 years old with a mean (SD) age of 22.66 (2.184). Table 1 gives the descriptive statistics for unadjusted and adjusted (Ehlers and Linear Models) IOP with and without fluorescein and for CCT for the participants. Apart from CCT, none of the parameters measured were normally distributed (Table 1).

## Results

**Figure 1:**
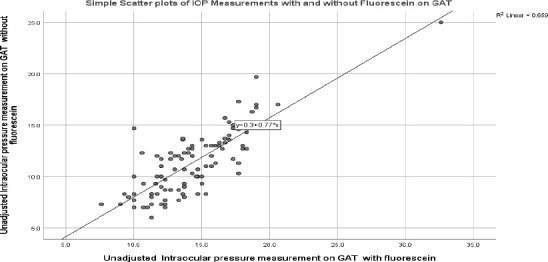
Scatterplot showing the correlation between IOP measurements with and without fluorescein on GAT

There was a significant positive correlation between CCT, and unadjusted IOP (r_s_ = 0.497, p < 0.0001) and adjusted IOP (r_s_ = 0.408, p < 0.0001). There was a statistically significant positive correlation between unadjusted IOP measurements with and without fluorescein with GAT (r_s_ = 0.76, p = 0.001) as indicated in Figure 1.

**Figure 2:**
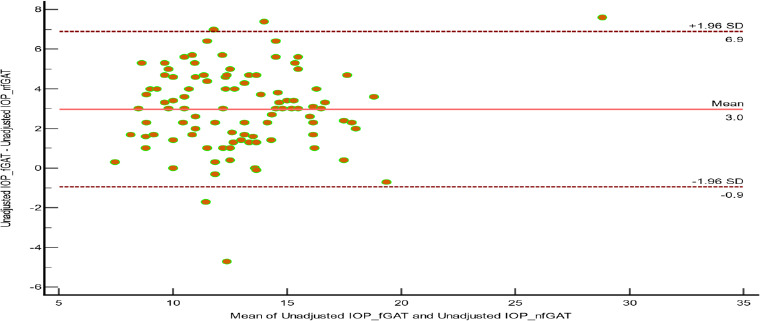
Bland Altman Scatter plot showing no agreement between unadjusted IOP measurements with fluorescein and without fluorescein

Similarly, the Intraclass Correlation Coefficient showed strong positive reliability between the IOP measurements with and without fluorescein with GAT, with an average measure of 0.728 (95% CI: -0.185 to 0.909). There was a significant median difference of 2.55mmHg between unadjusted IOP measurement with and without fluorescein with GAT, a mean difference of 2.97 (95% CI = 2.58 − 3.36) mmHg. Unadjusted IOP without fluorescein (MD 11.300) was significantly lower than unadjusted IOP with fluorescein (MD = 13.850) (Wilcoxon signed rank test, p < 0.001). There was no agreement between unadjusted IOP measurements with and without fluorescein with GAT using the Bland-Altman analysis [P < 0.0001, Lower limit: -0.9433, Upper limit: 6.8914; CI 95% = −1.6097 to -0.2769] (Figure 2).

There was no significant effect of gender on unadjusted IOP measurement with (Mann-Whitney U test, p = 0.110) and without (Mann-Whitney U test, p = 0.083) fluorescein; however, IOP measurement was relatively higher in males than in females. The Mean Rank for unadjusted IOP measurement with fluorescein was 57.23 and 47.77 in males and females, respectively. The Mean Rank for unadjusted IOP measurement without fluorescein was 57.63 and 47.38, respectively, for males and females.

### Relationship between Ehlers Adjusted IOP measurements and unadjusted IOP measurements, with and without Fluorescein on GAT

**Figure 3:**
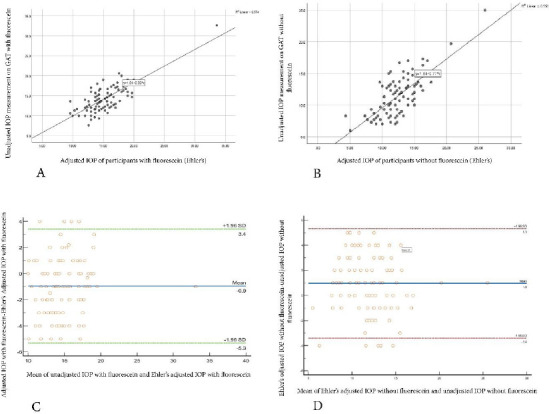
**A-** Scatterplot showing the correlation between unadjusted IOP measurements with fluorescein and Ehlers adjusted Model with fluorescein on GAT. **B-** Scatterplot showing the correlation between unadjusted IOP measurements without fluorescein and Ehlers adjusted Model without fluorescein on GAT. **C-** Bland Altman plot showing no agreement between Ehlers adjusted IOP and unadjusted IOP with fluorescein on GAT. **D-** Bland Altman plot showing no agreement between Ehlers adjusted IOP and unadjusted IOP without fluorescein on GAT

There was a statistically significant positive correlation between the Ehlers Adjusted IOP model and unadjusted IOP measurements with (r_s_ = 0.653, p < 0.0001) and without (r_s_ = 0.653, p < 0.0001) fluorescein. Ehlers' adjusted model with fluorescein showed relatively higher IOP measurements (MD = 15.00) in comparison to unadjusted IOP measurements (MD =13.85) with fluorescein (Wilcoxon signed rank test, p < 0.001). Similarly, Ehlers adjusted model without fluorescein showed comparatively high IOP measurements (MD = 12.30) in contrast to the unadjusted IOP without fluorescein (MD = 11.30) (Wilcoxon signed rank test, p < 0.001). There was no agreement between Ehlers adjusted IOP measurement and unadjusted IOP measurements with and without fluorescein on the Bland Altman analysis (p > 0.05) as indicated in Figure 3.

### Relationship between Linear Adjusted IOP measurements and unadjusted IOP measurements with and without fluorescein with GAT

**Figure 4:**
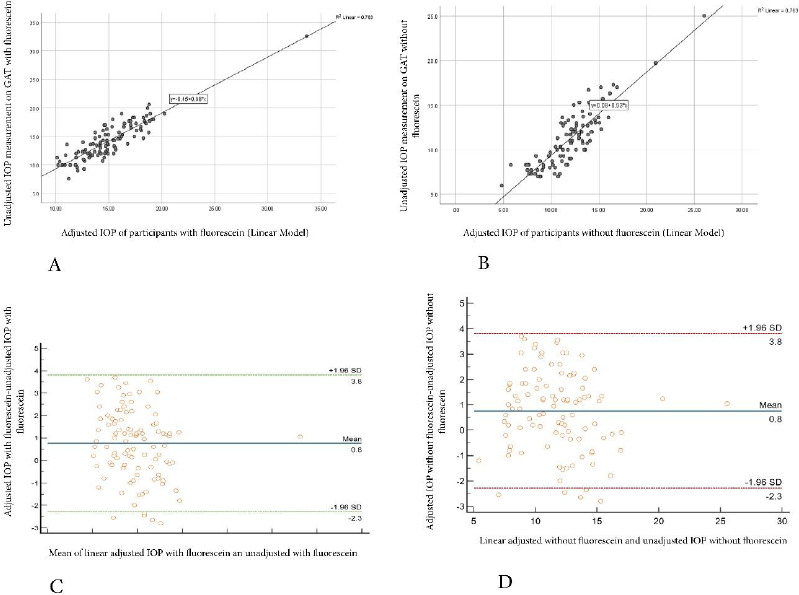
**A-** Scatterplot showing the correlation between unadjusted IOP measurements with fluorescein and Linear adjusted Model with fluorescein on GAT. **B-** Scatterplot showing the correlation between unadjusted IOP measurements without fluorescein and Linear adjusted Model without fluorescein on GAT. **C-** Bland Altman plot showing no agreement between Linear adjusted IOP and unadjusted IOP with fluorescein on GAT. **D-** Bland Altman plot showing no agreement between Linear adjusted IOP and unadjusted IOP without fluorescein on GAT

There was a statistically significant positive correlation between the Linear Adjusted IOP model and unadjusted IOP measurements with (r_s_ = 0.837, p < 0.0001) and without (r_s_ = 0.839, p < 0.0001) fluorescein, respectively. Linear adjusted model with fluorescein showed relatively higher IOP measurements (MD = 14.75) in comparison to unadjusted IOP measurements with fluorescein (MD = 11.85) (Wilcoxon signed rank test, p < 0.001). Similarly, the linear adjusted model without fluorescein showed comparatively high IOP measurements (MD = 12.00) in contrast to the unadjusted IOP without fluorescein (MD = 11.30) (Wilcoxon signed rank test, p < 0.001). There was no agreement between Linear adjusted IOP measurement and unadjusted IOP measurements with and without fluorescein using the Bland Altman analysis (p > 0.05) as summarised in Figure 4

## Discussion

The significantly lower unadjusted IOP without fluorescein compared to with fluorescein of 3.00 mmHg as found in this present study is clinically meaningful and may be a major contributing factor in influencing a practitioner's choice of diagnosis and management of glaucoma in Ghanaian populations, especially in borderline cases. This disagreement between the two techniques indicates that they cannot be used interchangeably in clinical diagnosis and monitoring treatment for glaucoma. It also signifies that measuring IOP using GAT without fluorescein underestimates the IOP in this Ghanaian population, as GAT with fluorescein is the gold standard.^12^,^13^ The specific danger with measuring IOP without fluorescein for this population is that eyes with higher IOPs will be at a greater risk of developing glaucomatous optic atrophy and visual field loss since IOPs will always be underestimated. This lower IOP for GAT without fluorescein compared to with fluorescein in this study is comparable with similar studies.^13^,^15^,^18^, Among the Sudanese population,^18^ GAT with fluorescein was 2.08 mmHg higher than that without fluorescein among participants; a higher difference of 3.41 mmHg was found comparing glaucomatous and non-glaucomatous populations.^18^ Another study^13^ found a clinically significant difference of approximately 7 mmHg, indicating that GAT without fluorescein is purely speculative. The difference, according to these authors^17^,^18^, may be attributed to the decreased predetermined applanation area of less than 3.06mm diameter when IOP is measured without fluorescein in GAT. This decreased pre-determined applanation area of less than 3.06mm, coupled with less distinct tonometer mires without fluorescein with GAT, may make it difficult for the examiner to visualise the edges of the flattened applanation area of the cornea and target, thus making it difficult to determine the correct application endpoint. Practitioners may stop too soon before reaching the standardised 3.06mm diameter, resulting in lower IOP measures. Also, considering the Imbert-Fick formula of Pressure = Force / Area, the reduced applanation area of less than 3.06mm may require the application of less force, ultimately resulting in lower IOP measurements.

Other studies, 12, 16,19, however, found contrasting results among some populations. A study^12^ on glaucomatous and non-glaucomatous Malawian populations found no significant difference between GAT with and without fluorescein; the study^12^ indicated that the evidence of GAT measurement with and without fluorescein is not hypothetical and emphasised that the two techniques can be used interchangeably in diagnosis and management of glaucoma among their population. Another study^16^ on healthy subjects from Turkey found no significant difference between IOP using GAT with and without fluorescein, emphasising that GAT without fluorescein was suitable for routine clinical practice. One study^19^ indicates that GAT without fluorescein can substitute GAT with fluorescein in the Indian population, helping to prevent the complications that come with fluorescein, and at the same time not compromising the gold standard. Based on the above population differences in results, the findings in the present study are limited to only Ghanaian populations within the age range of nineteen to twenty-nine years old.

The positive correlation between CCT and IOP is consistent with previous studies among juveniles in China^23^ and Indigenous Africans in Nigeria.^24^ The thin CCT (mean, 529.8µm) among the population studied is comparable to that reported in other African populations.^24^,^25^ Also, the correlation between unadjusted IOP measured with and without fluorescein was consistent with previous studies.^17^,^18^,^20^

Several tonometry correction factors have evolved in clinical practice to adjust IOP measurements in patients, considering factors that can erroneously influence the measurement. Widely used correction models include the Ehlers correction nomogram and Linear model, which predict the influence of CCT on IOP by generating a biomechanically adjusted estimate of the pressure^26^. These practices, currently used in many clinical settings, are not without limitations. The present study presented novel knowledge on the association between IOP readings on GAT with and without fluorescein using the Linear and Ehlers-adjusted models and the unadjusted IOP measurements with and without fluorescein.

Results from the study show that the IOPs with and without fluorescein using the Linear and the Ehlers models were significantly higher than their corresponding unadjusted IOP measurements. A study^27^ comparable to the present one recounts that correction models, such as the Ehlers models, overestimate the predicted effect of CCT on IOP measurements using GAT.

Clinicians should therefore take cautionary measures when using these correction factors in measuring patients' IOPs. A major practical limitation that influenced the minimal sample size was the limited availability of participants during the study period.

## Conclusion

We determined the agreement between IOP measurements with and without fluorescein on GAT. IOP measurements without fluorescein were significantly lower compared to IOP measurements with fluorescein with GAT; this difference was also clinically meaningful. This suggests that IOP measurement without fluorescein is not a better substitute for IOP measurement with fluorescein, especially in the Ghanaian population. Furthermore, IOP correction factor models, Ehlers and Linear, resulted in overestimated values compared to their corresponding unadjusted findings with and without fluorescein on GAT.
